# Critical Role of ADAMTS-4 in the Development of Sporadic Aortic Aneurysm and Dissection in Mice

**DOI:** 10.1038/s41598-017-12248-z

**Published:** 2017-09-27

**Authors:** Pingping Ren, Michael Hughes, Swapna Krishnamoorthy, Sili Zou, Lin Zhang, Darrell Wu, Chen Zhang, John A. Curci, Joseph S. Coselli, Dianna M. Milewicz, Scott A. LeMaire, Ying H. Shen

**Affiliations:** 1 Division of Cardiothoracic Surgery, Michael E. DeBakey Department of Surgery, Baylor College of Medicine, Houston, Texas USA; 2 Department of Cardiovascular Surgery, Texas Heart Institute, Houston, Texas USA; 3 Department of Vascular Surgery, Changzheng Hospital, Second Military Medical University, Shanghai, China; 4 Division of Vascular Surgery, Vanderbilt University School of Medicine, Nashville, Tennessee USA; 5 Division of Medical Genetics, Department of Internal Medicine, The University of Texas Health Science Center at Houston, Houston, Texas USA; 6 Cardiovascular Research Institute, Baylor College of Medicine, Houston, Texas USA; 7 Department of Molecular Physiology and Biophysics, Baylor College of Medicine, Houston, Texas USA

## Abstract

Sporadic aortic aneurysm and dissections (AADs) are common vascular diseases that carry a high mortality rate. ADAMTS-4 (a disintegrin-like and metalloproteinase with thrombospondin motifs-4) is a secreted proteinase involved in inflammation and matrix degradation. We previously showed ADAMTS-4 levels were increased in human sporadic descending thoracic AAD (TAAD) samples. Here, we provide evidence that ADAMTS-4 contributes to aortic destruction and sporadic AAD development. In a mouse model of sporadic AAD induced by a high-fat diet and angiotensin II infusion, ADAMTS-4 deficiency (*Adamts-4−/−*) significantly reduced challenge-induced aortic diameter enlargement, aneurysm formation, dissection and aortic rupture. Aortas in *Adamts-4−/−* mice showed reduced elastic fibre destruction, versican degradation, macrophage infiltration, and apoptosis. Interestingly, ADAMTS-4 was directly involved in smooth muscle cell (SMC) apoptosis. Under stress, ADAMTS-4 translocated to the nucleus in SMCs, especially in apoptotic SMCs. ADAMTS-4 directly cleaved and degraded poly ADP ribose polymerase-1 (a key molecule in DNA repair and cell survival), leading to SMC apoptosis. Finally, we showed significant ADAMTS-4 expression in aortic tissues from patients with sporadic ascending TAAD, particularly in SMCs. Our findings indicate that ADAMTS-4 induces SMC apoptosis, degrades versican, promotes inflammatory cell infiltration, and thus contributes to sporadic AAD development.

## Introduction

Aortic aneurysm and dissection (AAD) are common vascular diseases^1^. Aortic rupture frequently leads to death, particularly when the disease involves the thoracic aorta. Sporadic AADs, which account for more than 70% of cases, are caused by the progressive loss of smooth muscle cells (SMCs) and the destruction of extracellular matrix (ECM)^2,3^. Identifying molecules involved in these processes is critical for developing pharmacologic strategies to prevent AAD formation and progression.

Matrix proteases, such as matrix metalloproteinases (MMPs)^4^, play a critical role in the degradation of aortic ECM and in the formation of AAD. ADAMTSs (a disintegrin and metalloproteinase with thrombospondin motifs)^5–8^ are key extracellular metalloproteinases involved in ECM turnover. Similar to MMPs, ADAMTSs have been implicated in tissue destruction^9–11^ and inflammation^12,13^. We have recently reported the increased expression of ADAMTS-4 in aortic tissues from patients with descending thoracic AAD^14^. However, it is unclear whether ADAMTS-4 plays a role in AAD development.

In this study, we tested the hypothesis that ADAMTS-4 plays a critical role in AAD development. Specifically, we assessed the role of ADAMTS-4 in sporadic AAD development in mice, studied its effects on macrophage migration and SMC apoptosis in cultured cells, and examined its expression in aortic tissues from patients with sporadic ascending thoracic aortic aneurysm and dissection (aTAAD). Our findings show that ADAMTS-4 plays a critical role in sporadic AAD formation by degrading veriscan, promoting inflammatory cell infiltration, and inducing SMC apoptosis. Furthermore, ADAMTS-4 levels were significantly increased in sporadic TAAD.

## Results

### ADAMTS-4 expression is increased in mice with AAD

**Figure 1:**
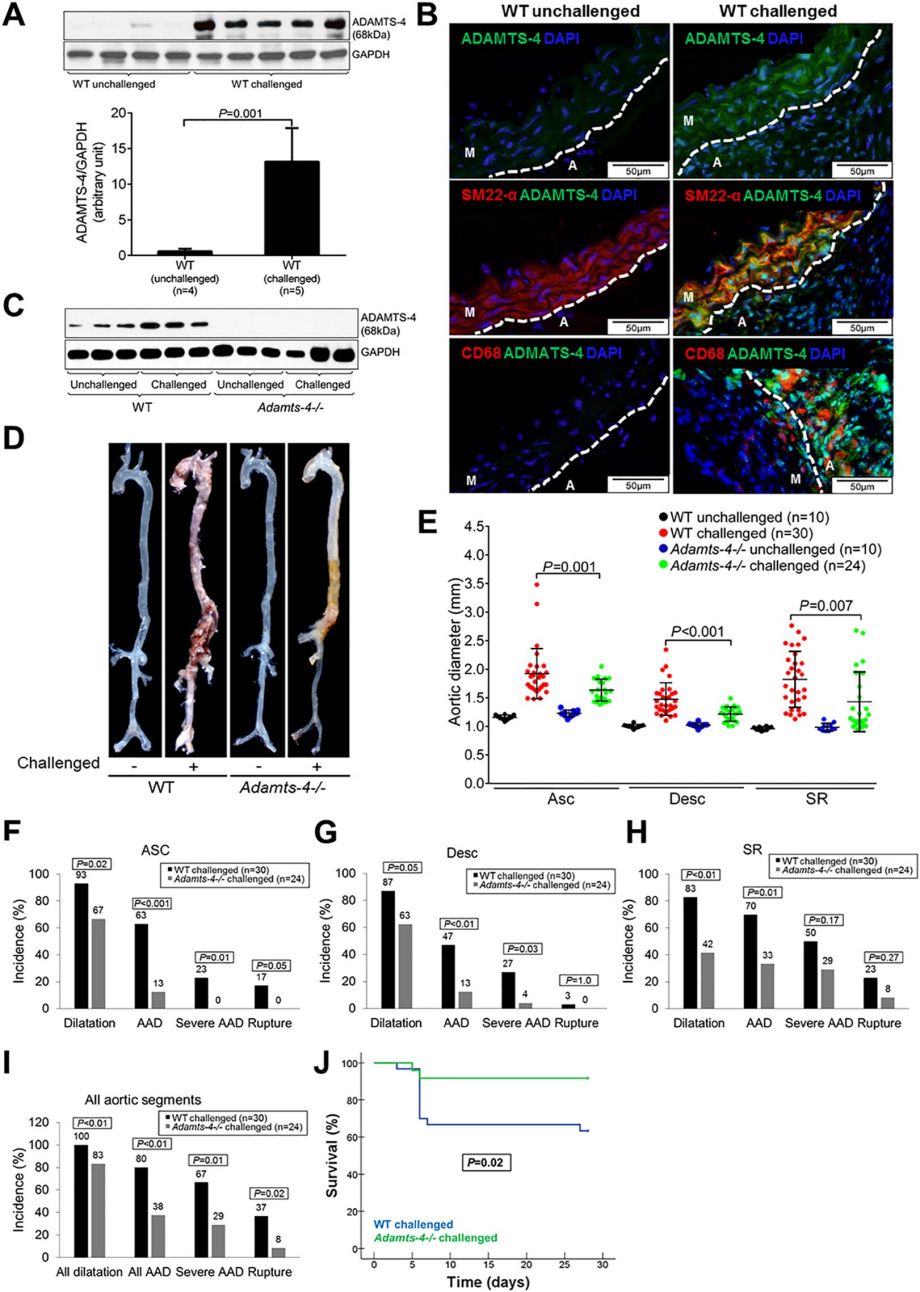
Reduced incidence of aortic aneurysm and dissection (AAD) in *Adamts-4−/−* mice. *Adamts-4−/−* and WT mice were unchallenged or challenged with a high-fat diet for 8 weeks and angiotensin II infusion (2000 ng/min/kg) during the last 4 weeks. (**A**) Representative images and quantification of western blot analysis of aortas from unchallenged WT mice (n = 4) and challenged WT mice (n = 5) showing the markedly increased expression of ADAMTS-4 in the aortas of challenged WT versus unchallenged WT mice. (**B**) Representative immunofluorescence staining images showing significant expression of ADAMTS-4 in SM22α+ SMCs and CD68+ macrophages in the aortas of challenged WT mice. (M: media; A: adventitia) **(C)** Representative images of western blots of aortas of unchallenged and challenged WT mice (n = 3) and unchallenged and challenged *Adamts-4−/−* mice (n = 3) showing no ADAMTS-4 protein expression in *Adamts-4−/−* mice. (**D**) Representative images of excised aortas showing less aortic damage in challenged *Adamts-4−/−* mice than in challenged WT mice. (**E**) Comparison of diameters in the ascending thoracic (Asc), descending thoracic (Desc), and suprarenal abdominal (SR) aortic segments and (**F-I**) the incidence of aortic dilatation, AAD, severe AAD, and aortic rupture in challenged WT and *Adamts-4−/−* mice. (**J**) Kaplan-Meier survival analysis showing survival in challenged *Adamts-4−/−* and WT mice at 28 days.

To determine the role of ADAMTS-4 in AAD development, we first examined ADAMTS-4 expression in the aortas of mice in which a sporadic AAD was induced by a high-fat diet and angiotensin II infusion^15^. ADAMTS-4 was barely detectable in the aortas of unchallenged mice but was significantly induced in the aortic tissue of challenged mice (Fig. 1A), especially in SMCs and macrophages in the aortic wall (Fig. 1B). Similarly, ADAMTS-1 was also increased in aortas from challenged mice (Fig. S1).

### ADAMTS-4 deficiency in mice significantly reduces AAD formation and rupture

We next examined the role of ADAMTS-4 in AAD development by comparing AAD formation in challenged wild-type (WT) mice and in challenged ADAMTS-4–deficient (*Adamts-4−/−*) mice that had no detectable ADAMTS-4 protein (Fig. 1C). As expected, challenge with a high-fat diet and angiotensin II infusion led to aortic destruction (Fig. 1D) and aortic enlargement (Fig. 1E) in WT mice. Importantly, aortic destruction was partially prevented (Fig. 1D) and aortic enlargement (Fig. 1E) was significantly reduced in challenged *Adamts-4−/−* mice as compared with challenged WT mice. Additionally, aortic challenge caused significant aortic dilatation (aortic diameter >1.25× the mean aortic diameter of unchallenged mice), aneurysm (aortic diameter >1.5× the mean aortic diameter of unchallenged mice), dissection (the presence of intramural thrombus), and rupture in thoracic and abdominal segments. The incidence of AAD (aortic aneurysm, dissection and rupture), severe AAD (dissection and rupture), and rupture was reduced in ascending (Fig. 1F), descending thoracic (Fig. 1G), and suprarenal abdominal (Fig. 1H) aortic segments. Furthermore, the overall incidence of aortic dilatation, AAD, severe AAD, and aortic rupture in all aortic segments was significantly reduced in challenged *Adamts-4−/−* mice as compared with challenged WT mice (Fig. 1I). Finally, we observed a significant increase in survival at 28 days in *Adamts-4−/−* mice compared with WT mice (Fig. 1J). Together, these data clearly indicate the critical role of ADAMTS-4 in AAD development in both thoracic and abdominal aortic regions.

### ADAMTS-4 deficiency in mice partially prevents aortic destruction and proteoglycan degradation

**Figure 2:**
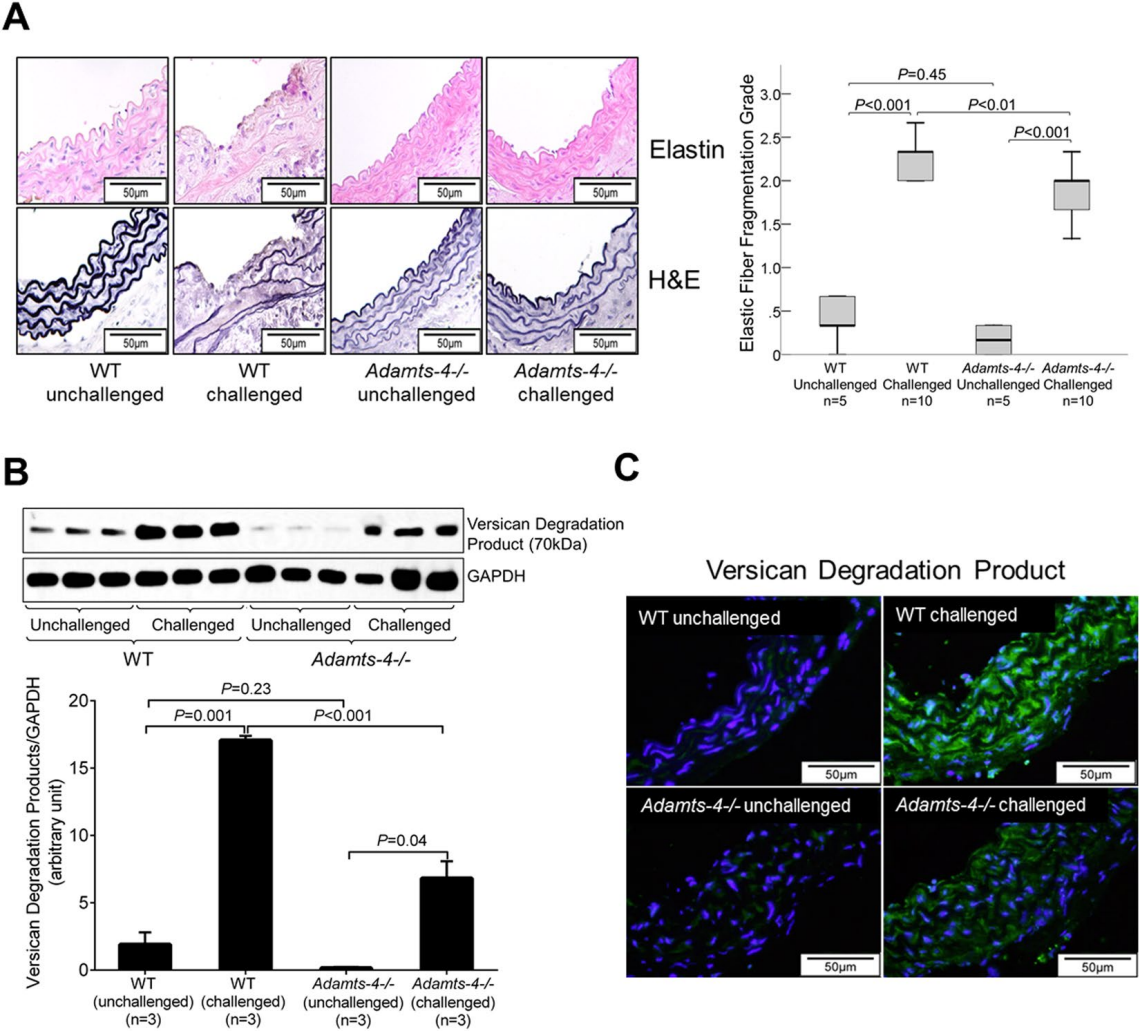
Decreased aortic elastic fibre destruction and proteoglycan degradation in *Adamts-4−/−* mice. (**A**) Representative images of hematoxylin and eosin (HE) staining and Verhoeff–van Gieson elastin staining (elastin) of ascending aortic sections from unchallenged and challenged WT and *Adamts-4−/−* mice. Quantification studies showing less elastic fibre fragmentation in aortas from challenged *Adamts-4−/−* mice than in aortas from challenged WT mice. Western blot analysis (**B**) and representative immunofluorescence staining images (**C**) indicating less versican degradation in aortas from challenged *Adamts-4−/−* mice than in aortas from challenged WT mice.

We further performed a histologic analysis of the aorta in challenged WT and *Adamts-4−/−* mice. Hematoxylin and eosin staining and Verhoeff–van Gieson elastic fibre staining showed severe destruction and elastic fibre fragmentation in the aortas of challenged WT mice (Fig. 2A). However, aortic destruction was reduced in *Adamts-4−/−* mice, suggesting the involvement of ADAMTS-4 in aortic destruction.

The main substrates of ADAMTS-4 are proteoglycans, which are important for vascular functions. Using an antibody that specifically detects degradation products of the proteoglycan versican, we found an increase in cleavage products in the aortas of challenged WT mice when compared with aortas of unchallenged mice (Fig. 2B and C). Moreover, the amount of degradation product was reduced in the aortas of challenged *Adamts-4−/−* mice (Fig. 2B and C), indicating involvement of ADAMTS-4 in versican degradation during AAD formation.

### ADAMTS-4 deficiency in mice decreases inflammatory cell infiltration in the aorta

**Figure 3:**
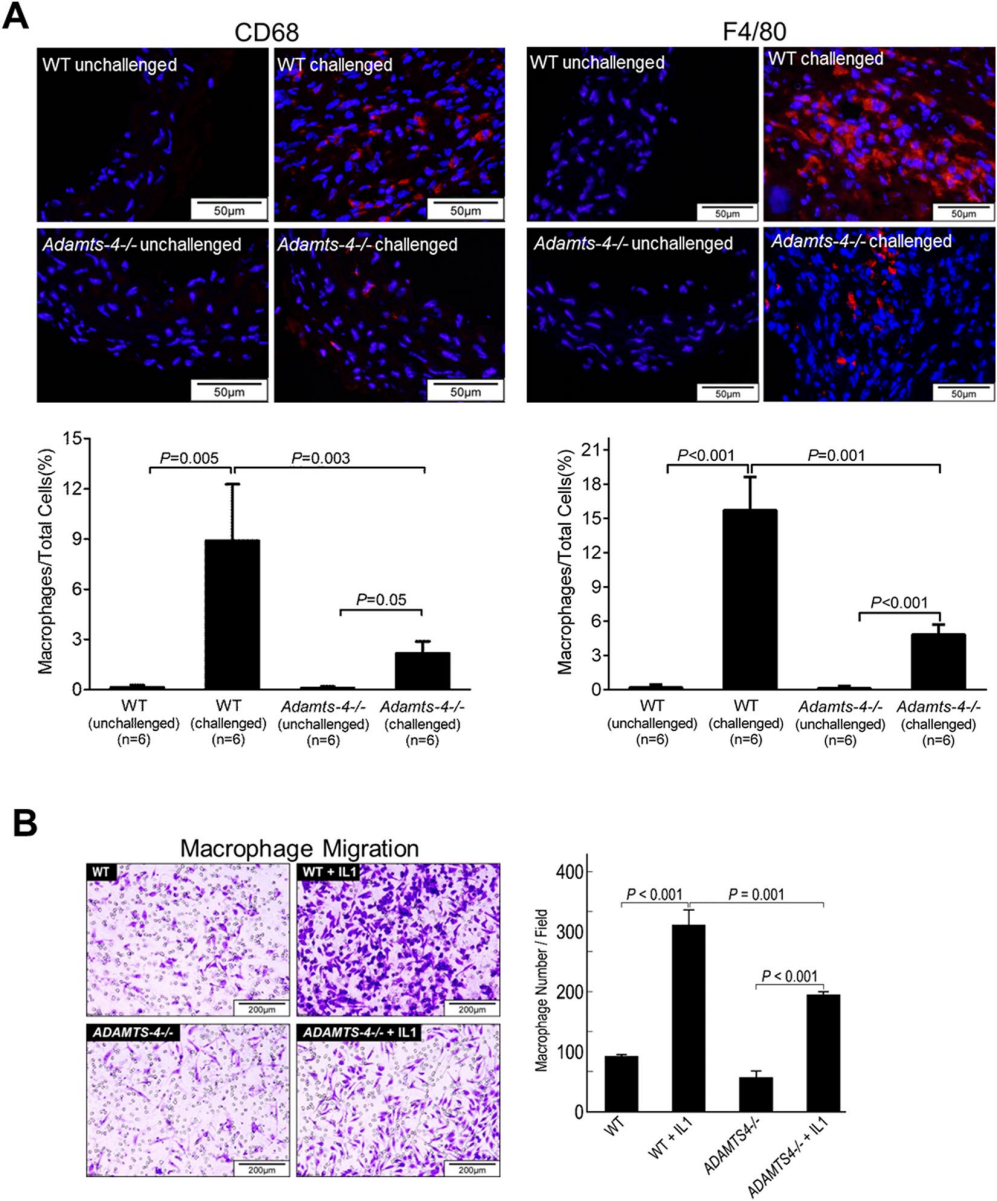
Decreased macrophage infiltration in the aortas of *Adamts-4−/−* mice. **(A)** Representative immunofluorescence staining images showing reduced CD68+ and F4/80+ macrophage infiltration in aortas from challenged *Adamts-4−/−* mice than in aortas from challenged WT mice. **(B)** Transwell assay indicating significantly less interleukin (IL)-1–induced invasion of macrophages from *Adamts-4−/−* mice than macrophages from WT mice.

Inflammatory cell infiltration in the aortic wall is a well-known trigger for ECM degradation and aortic injury that leads to AAD^16,17^. Therefore, we examined the presence of inflammatory cells in the aortas of challenged WT and *Adamts-4−/−* mice. Figure 3A shows that CD68+ and F4/80+ macrophages were highly abundant in the aortas of challenged WT mice, whereas significantly fewer CD68+ and F4/80+ macrophages were detected in the aortas of challenged *Adamts-4−/−* mice, possibly because of less inflammatory cell invasion.

To further examine whether ADAMTS-4 directly affects macrophage invasion, we examined the invasion ability of macrophages from *Adamts-4 −/−* mice and WT mice. Interleukin-1 (IL-1) stimulated macrophage invasion through the ECM-coated membrane (Fig. 3B). However, significantly less IL-1-induced invasion was observed in macrophages from *Adamts-4−/−* mice than in macrophages from WT mice (Fig. 3B), indicating a potential role of ADAMTS-4 in promoting macrophage invasion.

### ADAMTS-4 deficiency in mice reduces aortic apoptosis

**Figure 4:**
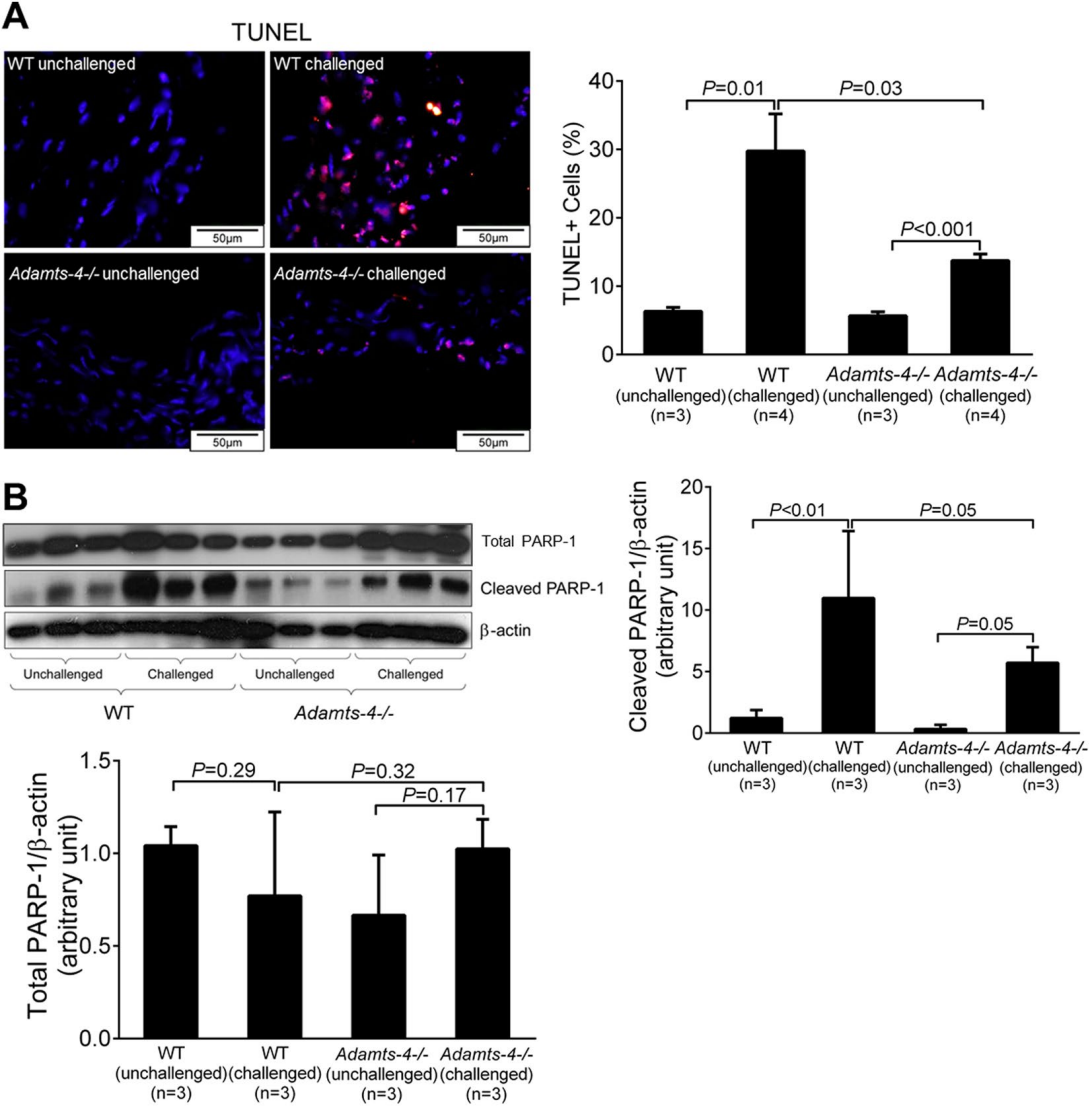
Decreased apoptosis in the aortas of *Adamts-4−/−* mice. (**A**) Representative images of TUNEL staining indicating less apoptosis in aortas from challenged *Adamts-4−/−* mice than in aortas from challenged WT. (**B**) Representative images of western blot analysis showing reduced PARP-1 cleavage products in the aorta of challenged *Adamts-4−/−* mice than in aortas from challenged WT mice but no difference in total PARP-1. Data shown are representative of 3 independent experiments.

Aortic cell apoptosis, a significant feature of AAD, may contribute to aortic destruction and disease development^18–20^. Increasing evidence suggests that ADAMTS-4 is involved in apoptosis^21,22^. We therefore examined the role of ADAMTS-4 in aortic apoptosis. As shown in Fig. 4A, TUNEL-positive cells were abundant in the aortic wall of challenged WT mice but were significantly reduced in the aortic wall of challenged *Adamts-4−/−* mice. Consistent with these results, we observed decreased cleavage of PARP-1, an important molecule involved in DNA repair and cell survival, in the aortas of challenged *Adamts-4−/−* mice when compared with the aortas of challenged WT mice (Fig. 4B). Together, these findings suggest the potential involvement of ADAMTS-4 in apoptosis during AAD formation.

### ADAMTS-4 is directly involved in SMC apoptosis

**Figure 5:**
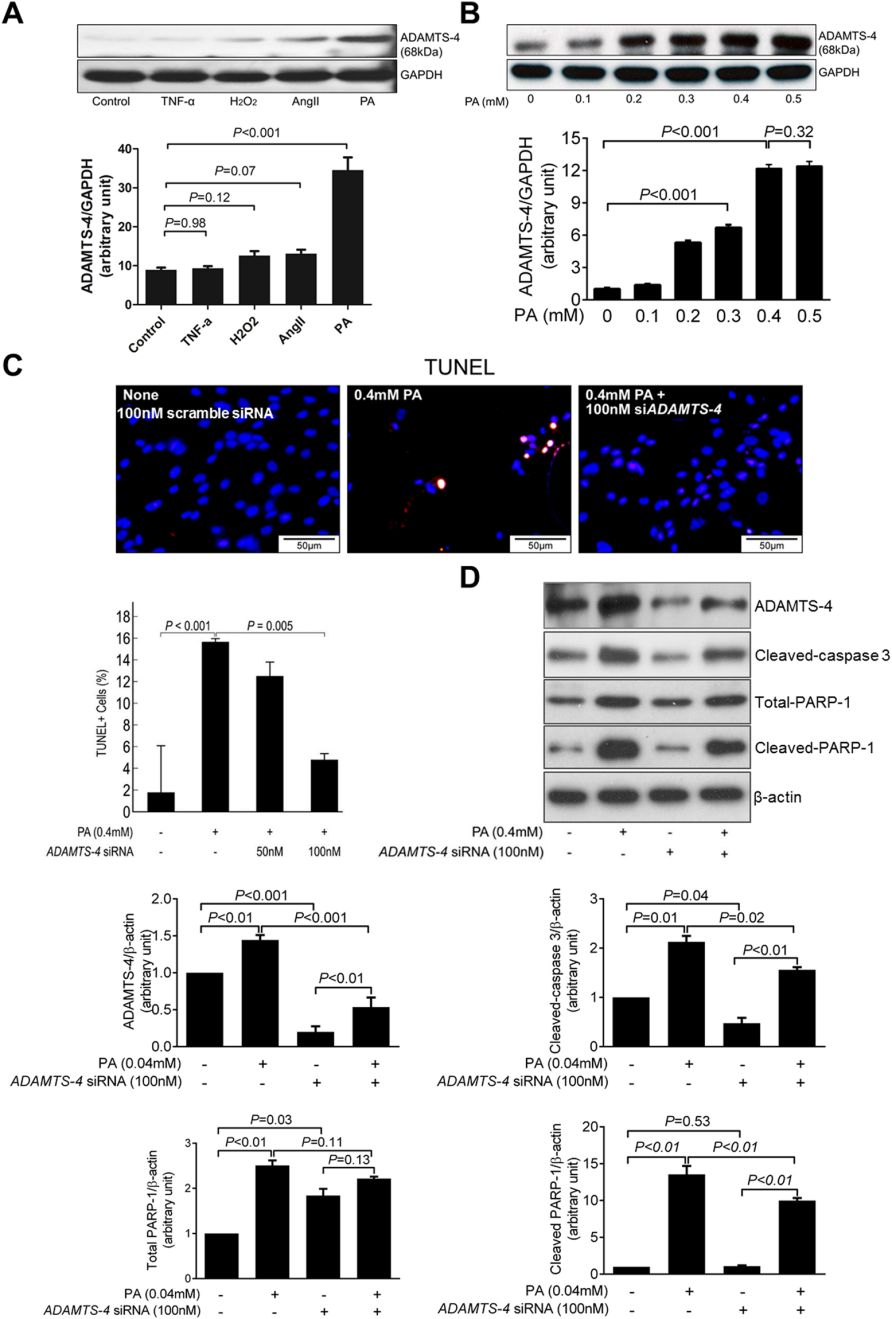
Involvement of ADAMTS-4 in smooth muscle cell apoptosis. (**A**) Western blot analysis of ADAMTS-4 in smooth muscle cells (SMCs) treated with tumor necrosis factor-α (TNF-α), hydrogen peroxide (H_2_O_2_), angiotensin II (AngII), or palmitic acid (PA). Quantification analysis showing that PA significantly induced ADAMTS-4 expression in SMCs. (**B**) SMCs treated with different dose of PA. Quantification of western blot analysis showing that PA induced ADAMTS-4 expression in a dose-dependent fashion. **(C)** Representative images and quantification of TUNEL staining show that PA induced SMC apoptosis and that the PA-induced apoptosis can be prevented by ADAMTS-4 siRNA. **(D)** Representative images of western blots showing that the levels of ADAMTS-4, cleaved-caspase-3 levels, and PARP-1 cleavage were reduced in ADAMTS-4 siRNA–transfected cells. Data shown are representative of 3 independent experiments.

Among the potential mechanisms by which ADAMTS-4 contributes to aortic destruction, we focused on its role in aortic apoptosis. Although ADAMTS-4 has been shown to participate in apoptosis^21,22^, the underlying mechanisms are poorly understood. ADAMTS-4 may promote apoptosis by degrading versican, which has been shown to inhibit apoptosis^23–26^. However, ADAMTS-4 may have a more direct role in apoptosis. To examine this possibility, we first tested several agents for their potency in stimulating the induction of ADAMTS-4 expression, including palmitic acid (PA), tumour necrosis factor-α, angiotensin II, and H_2_O_2_. PA was the most potent stimuli (Fig. 5A) that induced a dose-dependent increase in ADAMTS-4 (Fig. 5B). Additionally, PA mimics in cells the elevated serum free fatty acid levels in our sporadic AAD model (Fig. S2). Thus, we used PA in these studies as a trigger to induce ADAMTS-4 expression in human thoracic aortic SMCs. We showed that PA induced apoptosis (Fig. 5C) and the cleavage of PARP-1 (Fig. 5D), both of which were partially prevented by *ADAMTS-4* siRNA, indicating that ADAMTS-4 may directly promote SMC apoptosis.

### ADAMTS-4 translocates to the nucleus of apoptotic cells

**Figure 6:**
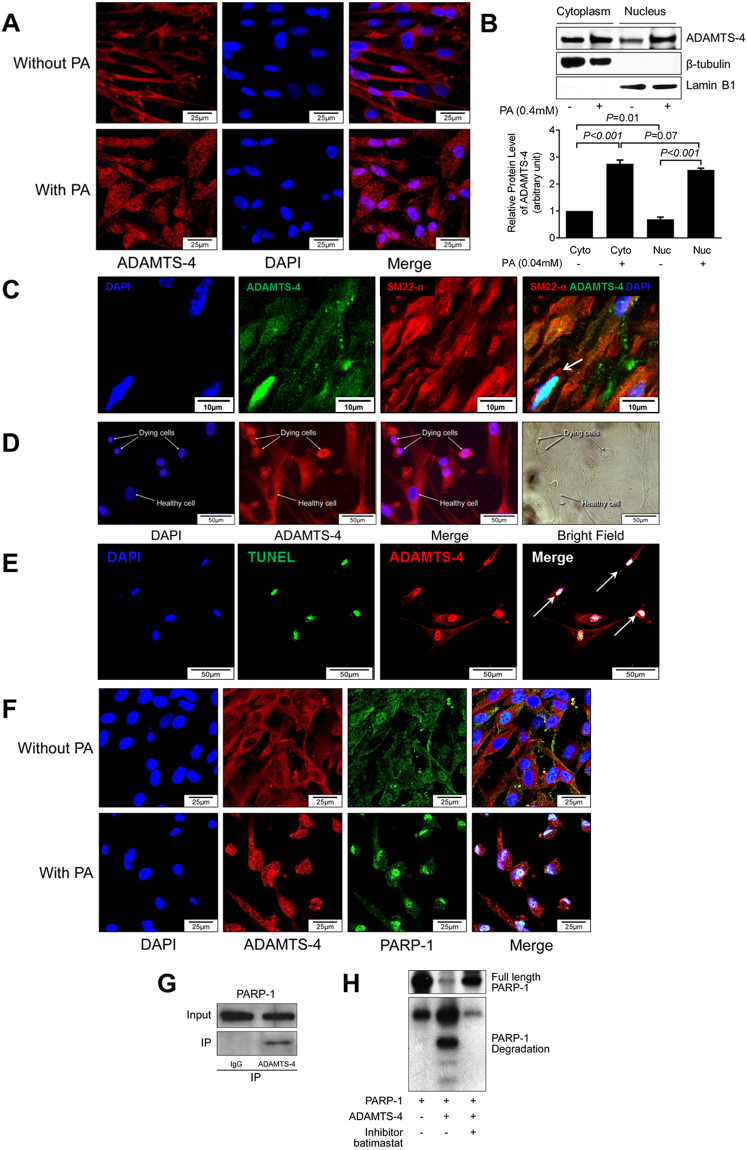
ADAMTS-4 moves to the nucleus and directly cleaves PARP-1. Representative images of confocal microscopy of immunofluorescence immunostaining **(A)** and western blot analysis **(B)** showing that PA induced ADAMTS-4 nuclear translocation. (**C**) Representative images of double-staining of ADAMTS-4 and SM22-α showing that ADAMTS-4 was seen in the nuclei of SMCs. (**D**) Representative confocal microscopy images of immunofluorescence staining showing that ADAMTS-4 was highly abundant in the condensed nuclei of dying cells. (**E**) Representative images of double-staining of ADAMTS-4 and TUNEL in PA treated SMCs showing ADAMTS-4 in the nuclei of TUNEL+ apoptotic cells.(**F**) Representative confocal microscopy images of immunofluorescence staining showing that ADAMTS-4 colocalized with PARP-1 in the nuclei of PA-treated cells. **(G)** Co-immunoprecipitation analysis indicating that ADAMTS-4 and PARP-1were in the same complex in SMCs. (**H**) Recombinant human PARP-1 was incubated with affinity-purified recombinant human ADAMTS-4 in the presence or absence of ADAMTS-4 inhibitor. Western blot analysis showing that ADAMTS-4 directly cleaved PARP-1. Data shown in (**A**–**F**) are representative of 3 independent experiments.

To further understand the direct role of ADAMTS-4 in PARP-1 cleavage and apoptosis, we studied the cellular location of ADAMTS-4. Immunostaining showed that ADAMTS-4 was localized primarily in the cytoplasm (Fig. 6A) in cells not exposed to PA, whereas a significant amount of ADAMTS-4 was detected in the nucleus of PA-treated cells (Fig. 6A). Western blot analysis confirmed that ADAMTS-4 was present in the nuclear fraction and that the levels of nuclear ADAMTS-4 were increased in PA-treated cells (Fig. 6B). Consistent with our findings in cultured cells, ADAMTS-4 was also detected in the nuclei of SMCs in diseased aortas in our mouse AAD model (Fig. 6C). Furthermore, while ADAMTS-4 was present in the cytoplasm of healthy SMCs, it was highly abundant in the nucleus of apoptotic SMCs (ie, cells with condensed nuclei) (Fig. 6D). Co-immunofluorescence staining showed the presence of ADAMTS-4 in the nuclei of TUNEL+ apoptotic cells (Fig. 6E). These findings indicate that ADAMTS-4 may translocate to the nucleus when SMCs are under stress.

### ADAMTS-4 directly interacts with and cleaves PARP-1

PARP-1 is a nuclear protein that plays a critical role in DNA repair and cell survival^27,28^. We therefore examined whether ADAMTS-4 can induce apoptosis by targeting PARP-1. Double immunofluorescence staining showed that ADAMTS-4 colocalized with PARP-1 in the nucleus of PA-treated SMCs (Fig. 6F). Co-immunoprecipitation experiments showed that ADAMTS-4 interacted with PARP-1 in PA-treated SMCs (Fig. 6G). Finally, by using a direct cleavage assay, we demonstrated that ADAMTS-4 can directly cleave PARP-1. Incubating recombinant PARP-1 with recombinant ADAMTS-4 (Fig. 6H) markedly increased PARP-1 cleavage and reduced full-length PARP-1. The PARP-1 cleavage was partially prevented by a pan ECM protease inhibitor. Together, our data suggest that, in response to stress, the ECM protease ADAMTS-4 can translocate to the nucleus and directly cleave/degrade PARP-1, which may result in impaired DNA repair and lead to SMC apoptosis.

### ADAMTS-4 expression is increased in human sporadic aTAAD tissue

**Figure 7:**
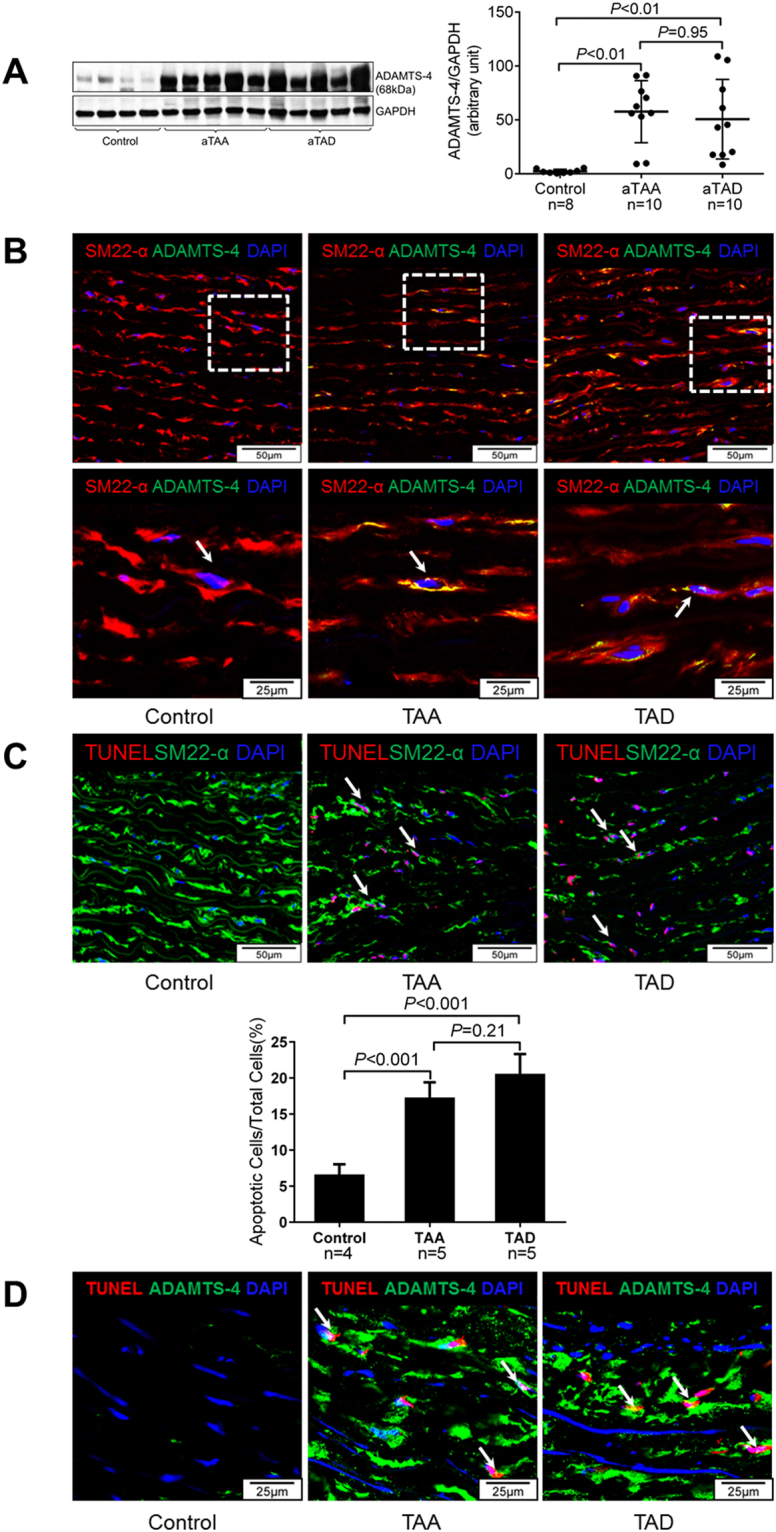
Increased expression of ADAMTS-4 in human sporadic aTAAD aortic tissue. (**A**) Ascending aortic tissues from patients with aTAA (n = 10) or acute aTAD (n = 10) and from organ donors (ascending control, n = 8) were analysed. Representative western blot images and quantification showing significant ADAMTS-4 expression in the aortic wall of aTAA and aTAD patients. (**B**) Representative images of immunofluorescence staining of ascending aortic sections illustrating that ADAMTS-4 was increased in SMCs in the aortas of patients with aTAA and aTAD. **(C)** Representative images and quantitative analysis of TUNEL staining of ascending aortic sections showing significant apoptotic cells in aortas of aTAA (n = 5) and aTAD (n = 5) patients, but not in control samples (n = 4). (**D**) Representative images of double-staining of ADAMTS-4 and TUNEL in human aortic tissue showing ADAMTS-4 in the nuclei of TUNEL+ apoptotic cells.

Finally, we examined ADAMTS-4 expression in aortic tissues from patients with sporadic ascending thoracic aortic aneurysm (aTAA) and dissection (aTAD). ADAMTS-4 levels were significantly higher in aortic tissues from aTAA and aTAD patients than in ascending aortic tissue from age-matched organ donors (controls) (Fig. 7A). ADAMTS-4 was present in SMCs in the medial layer of aTAAD tissues (Fig. 7B). We also detected TUNEL+ apoptotic SMCs (Fig. 7C) and the presence of ADAMTS-4 in the nuclei of TUNEL+ apoptotic cells (Fig. 7D) in diseased tissues. These findings indicate significant upregulation of ADAMTS-4 and its potential association with SMC apoptosis in sporadic aTAAD patient tissues.

## Discussion

In this study, we provide evidence that ADAMTS-4 plays an important role in aortic destruction and AAD formation. Using a mouse model of sporadic AAD, we have shown that *Adamts-4* deficiency significantly reduced aortic enlargement, AAD formation, and aortic rupture in both thoracic and abdominal segments. *Adamts-4* deficiency also reduced aortic destruction, versican degradation, inflammatory cell infiltration, and SMC apoptosis. In cultured SMCs, we found that ADAMTS-4 moved into the nucleus, directly cleaved PARP-1, and induced SMC apoptosis. Finally, we found significant expression of ADAMTS-4 in aortic tissue samples from patients with aTAAD. Together, our results suggest that ADAMTS-4 plays a critical role in the development of AAD.

The ADAMTS enzyme group has been implicated in inflammation and tissue destruction in cancer metastasis^9–11^ and in inflammatory diseases such as arthritis^12,13^. Several studies have suggested that the dysregulation of ADAMTS-4 may also contribute to the development of vascular diseases. For example, Wagsater and colleagues^29^ showed that ADAMTS-4 is highly expressed in macrophage-rich areas of human atherosclerotic plaque and may be involved in atherosclerotic plaque formation and stability. We and others^14,30^ have shown that ADAMTS-4 levels are increased in aortic tissues from patients with sporadic thoracic AAD. Here, we provide evidence that *Adamts-4* deficiency in mice reduced challenged-induced aortic destruction, aortic enlargement, and AAD formation and rupture. Our study establishes a critical role for ADAMTS-4 in AAD development and indicates that ADAMTS-4 may be a potential target for TAAD treatment.

ADAMTS-4 may induce aortic destruction through several mechanisms. ADAMTS-4 degrades proteoglycans, which are ECM components. Versican^21^, the most studied target of ADAMTS-4, plays an important role in maintaining vascular structural and functional integrity^23^ by retaining water, creating reversible compressive structures^24^, regulating elastic fibre assembly, inhibiting cell apoptosis, and stimulating cell growth and angiogenesis^24–27^. Degradation of versican by ADAMTS-4 may be an underlying mechanism for its contribution to vascular diseases^23^. We recently observed an increase in versican degradation in human descending thoracic AAD tissue samples, and versican degradation was correlated with increased ADAMTS-4 protein levels^16^. In our current study, we showed significantly reduced versican degradation products in *Adamts-4−/−* mice, suggesting that ADAMTS-4–mediated versican degradation may be partially responsible for aortic destruction and AAD formation.

Our findings also suggest that ADAMTS-4 may induce AAD formation by promoting aortic inflammation. Inflammatory cells such as macrophages play a critical role in the progression of aortic aneurysms^31^. Extracellular matrix proteases such as MMPs have been shown to promote inflammatory cell infiltration by digesting ECM^31^. Similarly, ADAMTS-1 is capable of stimulating cell invasion during cancer metastasis by digesting ECM^32–34^. Consistent with previous findings^15^, we detected an abundance of macrophages in the aortic wall of challenged WT mice. Importantly, inflammatory cell infiltration was significantly lower in challenged *Adamts-4−/−* mice than in challenged WT mice. Although it is possible that the reduced inflammatory infiltration was due to less aortic injury and inflammatory cell attraction in challenged *Adamts-4−/−* mice, our findings suggest that the reduced infiltration may be due to decreased inflammatory cell invasion. *Adamts-4−/−* macrophages showed reduced ECM invasion in response to IL-1 stimulation, indicating that ADAMTS-4 may digest ECM and promote macrophage infiltration.

Our study suggests that ADAMTS-4 may also contribute to AAD by promoting aortic SMC apoptosis, which has been associated with AAD development^18–20^. The mechanisms underlying the potential role of ADAMTS-4 in apoptosis^21,22^ are not well understood, but one potential mechanism may involve ADAMTS-4–induced degradation of versican, which functions to inhibit apoptosis^21,23–26^. Our novel findings indicate that ADAMTS-4 may directly induce apoptosis in SMCs. We showed that the siRNA-mediated knockdown of *ADAMTS-4* in PA-treated SMCs reduced the activation of apoptosis pathways and apoptotic cell death. These results support those of a recent study that also showed a direct role for ADAMTS-4 in apoptosis^22^. Thus, ADAMTS-4 may promote apoptosis by degrading versican or by directly inducing apoptosis in SMCs.

When we further investigated the mechanisms by which ADAMTS-4 directly induces SMC apoptosis, we found that ADAMTS-4 is translocated from the cytoplasm into the nucleus in the presence of PA, indicating a potential nuclear function of this extracellular protease. In addition, when we searched for potential targets of ADAMTS-4 in the nucleus, we found that ADAMTS-4 directly bound and cleaved PARP-1, which modulates protein functions^27,28,35^ by adding poly (ADP ribose) to its targets. Activated by various cellular stress signals^27^, PARP-1 plays a critical role in regulating many cellular functions, including cell metabolism^36^ and inflammation^35^. The most critical function of PARP-1 is detecting and repairing DNA damage^27,28^, a process that is necessary for cell survival. Moreover, increasing evidence has suggested that PARP-1 also promotes cell death^27,28,35^. The switch between PARP-1–mediated cell survival or death is partially controlled by its cleavage^37^; full-length PARP-1 promotes cell survival, whereas cleaved PARP-1 can induce cell death^37^. By directly cleaving PARP-1, ADAMTS-4 may prevent PARP-1–mediated DNA repair and cell survival and induce apoptosis. A recent study has suggested that the catalytic activity of ADAMTS-4 may not be required for apoptosis, as the catalytically inactive full form and the C-terminal domain of ADAMTS-4 could both increase apoptosis^22^. Further studies are necessary to understand whether ADAMTS-4 induces apoptosis through both catalytic activity–dependent and independent mechanisms and what the relationship is between these mechanisms.

Other mechanisms may also be responsible for the contributions of ADAMTS-4 to vascular tissue destruction. ADAMTS-1 inhibits cell proliferation by binding and sequestering growth factors, such as vascular endothelial growth factor and fibroblast growth factor^32^. ADAMTS-4 may act in a similar fashion by inhibiting SMC growth and thus preventing aortic repair, although this possibility requires further investigation. In addition, questions remain about what causes the increase in ADAMTS-4 levels in AAD and how to prevent ADAMTS-4 induction and activation. Future studies aim to identify the factors and pathways that promote ADAMTS-4 induction. Finally, because of its role in AAD development, ADAMTS-4 may serve as a therapeutic target. Several recently developed ADAMTS inhibitors have been shown to prevent inflammation and tissue destruction in a rat model of arthritis^38–41^. The potential use of ADAMTS-4 inhibitors in treating AAD warrants investigation. However, ADAMTS isoforms play diverse and possibly opposite roles in AAD development as has been shown by a recent study suggesting that ADAMTS-1 may be an important mediator of vascular wall homeostasis^42^. Thus, in considering the therapeutic use of ADAMTS-4, it is important to develop isoform-specific inhibitors rather than non-specific ADAMTS inhibitors for AAD prevention/treatment.

In conclusion, this study indicates that ADAMTS-4 plays an important role in AAD formation by promoting ECM destruction, inflammation, and apoptosis in the aorta. Furthermore, ADAMTS-4 can translocate into the nucleus, interact with and cleave PARP-1, and directly induce apoptosis. Future studies are needed to examine whether the pharmacologic inhibition of ADAMTS-4 prevents aortic destruction and AAD progression.

## Materials and Methods

### Patient enrolment

The protocol for collecting human tissue samples was approved by the institutional review board at Baylor College of Medicine. Informed consent forms were signed by all participants before enrolment. All experiments conducted on human tissue samples were performed in accordance with the relevant guidelines and regulations. For this study, we enrolled patients with sporadic aTAA (n = 10) and acute aTAD (n = 10) who were undergoing elective open operation to replace the diseased aorta with a graft. During aneurysm repair, we routinely excise tissue from the anterior-lateral portion of the aortic wall (the outer wall of the false lumen in dissection cases) in the region of the largest aortic diameter; a portion of this discarded tissue was collected for this study. Patient characteristics are shown in Table 1. In addition, we used aortic tissues from age-matched organ donors without aortic aneurysm, dissection, coarctation, or previous aortic repair as control samples (International Institute for the Advancement of Medicine, Jessup, PA, USA). To minimize aortic damage due to prolonged ischemia, we selected donors with a cardiac arrest time of less than 60 minutes; the aortas were collected within 60 minutes of termination of life support and were preserved in UW Belzer solution and shipped to our lab on wet ice. The time from control aorta collection to tissue processing/banking was <24 hours. Periaortic fat and intraluminal thrombus were trimmed from all aortic samples. The samples were rinsed with cold 0.9% normal saline and then divided; one portion was immediately snap-frozen in liquid nitrogen and stored at –80 °C for protein extraction, and the other portions were embedded in optimal cutting temperature (OCT) compound for immunofluorescence staining.

**Table 1:** Patient characteristics.

Characteristics	Control (n = 8)	aTAA (n = 10)	aTAD (n = 10)
Age (y)	63.9 ± 8.4	66.5 ± 8.9	62.9 ± 9.0
Male	4 (50%)	5 (50%)	6 (60%)
Hypertension	6 (75%)	10 (100%)	10 (100%)
COPD	1 (13%)	1 (10%)	1 (10%)
Diabetes mellitus	2 (25%)	2 (20%)	2 (20%)
History of smoking	3 (38%)	4 (40%)	6 (60%)
Use of anti-lipid medication	2 (25%)	2 (20%)	1 (10%)
Use of COX inhibitor	3 (38%)	6 (60%)	5 (50%)
Aortic diameter (cm)	NA	6.0 ± 0.9	5.4 ± 0.8

Data are expressed as a number (percent) or as the mean ± standard deviation. aTAA, ascending thoracic aortic tissue from patients with ascending thoracic aortic aneurysm; aTAD, ascending thoracic aortic tissue from patients with acute ascending thoracic aortic dissection; COPD, chronic obstructive pulmonary disease; COX, cyclooxygenase; NA, not available.

### Mouse study design

All mice were bred and maintained in the animal care facility at Baylor College of Medicine, and all animal procedures were conducted according to the approved guidelines of the Institutional Animal Care and Use Committee (IACUC) of Baylor College of Medicine in accordance with the Guide for the Care and Use of Laboratory Animals, published by the US National Institutes of Health. All animal experimental protocols described in the manuscript have been approved by the IACUC (protocol number AN-4195). WT (C57BL/6 J) and ADAMTS-4–deficient mice (*Adamts-4−/−*) in C57BL/6 J background were purchased from The Jackson Laboratory (Bar Harbor, ME, USA).

We challenged 4-week-old male WT mice (n = 30) and *Adamts-4−/−* mice (n = 24) with a high-fat diet for 8 weeks and infused 2,000 ng/min/kg angiotensin II (Sigma-Aldrich Corp., St. Louis, MO, USA) during the last 4 weeks through an osmotic minipump (Model 2004; ALZA Scientific Products, Mountain View, CA, USA). The high-fat diet (Research Diets, Inc., D12108C, New Brunswick, NJ, USA) contained 20% protein, 40% carbohydrate, 40% fat, and 1.25% cholesterol. We fed 4-week-old WT mice (n = 10) and *Adamts-4−/−* mice (n = 10) a chow diet for 8 weeks and infused them with saline during the last 4 weeks as unchallenged controls.

At the end of the 8-week study period, mice were euthanized, and their aortas were irrigated with cold phosphate-buffered saline (PBS). Aortas were embedded in OCT compound for histology and immunofluorescence staining or were snap-frozen for protein analysis. Serum samples were collected, and the level of free fatty acids in the serum was detected by using commercially available kits (Abcam) according to the manufacturer’s instructions.

### Criteria for aortic aneurysm, dissection, and rupture

We measured the diameter of the ascending, arch, and descending thoracic aortic segments of the extracted aortas. In euthanized mice, we exposed and rinsed the aorta with cold PBS and removed the periaortic tissues. The aorta was excised and further cleaned and rinsed with cold PBS to remove any residual blood in the lumen. Images of the aorta were obtained using an Olympus SZX7 microscope at a magnification of 0.4X (scale bar 2mm), and the diameter of each aortic segment was measured with DP2-BSW software (Olympus Life Science Solutions, Center Valley, PA, USA) by two independent observers who were blinded to the animal group. The mean aortic diameters of unchallenged WT mice (n = 10) and *Adamts-4−/−* mice (n = 10) served as a baseline diameter to determine aortic aneurysm formation in challenged mice. The mean diameter of the different regions was calculated and compared among the groups. For each aortic segment, dilatation in challenged WT or *Adamts-4−/−* mice was defined as an aortic diameter ≥1.25 but <1.5 times the mean aortic diameter of the segment in unchallenged mice with the same genetic background; aneurysm in challenged WT or *Adamts-4−/−* mice was defined as an aortic diameter ≥1.5 times the mean aortic diameter of the segment in unchallenged mice with the same genetic background. We defined aortic dissection as the presence of a tear in the aortic media or in the media-adventitia boundary with the presence of intramural thrombus or a false lumen hematoma in an aortic cross section. Aortic rupture and premature death were documented.

### Severity of aortic aneurysm and dissection

The gross appearance of each aorta was assessed for severity of AAD formation according to a scheme based on the classification system described by Daugherty and colleagues^20^: aortic dilatation (aortic diameter ≥1.25 but <1.5 times the aortic diameter of unchallenged mice with the same genetic background); aortic aneurysm (aortic diameter ≥1.5 times the aortic diameter of unchallenged mice with the same genetic background); and dissection (indicated by intramural thrombus). We further defined AAD (includes aortic aneurysms, dissection and rupture) and severe AAD (includes aortic dissection and aortic rupture). Each aorta was evaluated independently by 2 observers blinded to the animal group. In cases of discrepancies, the observers discussed the cases and reached an agreement on the classification.

### Analysis of aortic structure

Paraffin-embedded aortic sections were subjected to haematoxylin and eosin staining and Verhoeff–van Gieson elastin staining (Sigma-Aldrich) according to the manufacturer’s instructions. Two independent observers who were blinded to the animal group allocation examined 3 aortic sections per aorta from 5–10 mice per group. The extent of elastic fibre fragmentation was scored on a scale of 0 to 3 (grade 0 = none, grade 1 = minimal, grade 2 = moderate, and grade 3 = severe).

### Immunofluorescence staining

For immunofluorescence staining, OCT-embedded aortic sections or treated cells were fixed with Cytofix (BD Biosciences, San Jose, CA, USA) and were permeabilized with Perm/Wash (BD Biosciences). Nonspecific staining was reduced by blocking with 10% normal blocking serum. Sections were stained with primary antibodies against CD68 (ab955, Abcam, USA), ADAMTS-4 (ab28285, Abcam), SM22-α (ab10135, Abcam), versican degradation product (ab19345, Abcam), or PARP-1 (9548, Cell Signaling, USA), at 4 °C overnight and then stained with secondary antibodies conjugated to an Alexa Fluor dye (Alexa Fluor 568 or 488; Invitrogen, Carlsbad, CA, USA). Nuclei were counterstained with 4′,6-diamidino-2-phenylindole (DAPI). Sections or cells were examined by using a Leica SP5 confocal microscope (Leica Microsystems Inc., Wetzlar, Germany) or an Olympus (Tokyo, Japan) immunofluorescence microscope. For quantification, we randomly selected 4–5 fields (40X magnification) per tissue section. The same exposure time was used for all samples. The mean signal intensity as normalized to the aortic area was compared among the groups.

### Macrophage invasion assay

Peritoneal macrophages were harvested from WT and *Adamts-4−/−* mice as previously described^17^. Flow cytometry confirmed that 87% of the cells were macrophages. Macrophage viability was assessed by the MTT cell viability assay. Macrophage invasion was measured by using a modified Boyden chamber assay with an 8-μm pore size membrane coated with a thin layer of ECMatrix as a barrier (Chemicon International, Billerica, MA, USA). Macrophages (2.5 × 10^5^) were added to the upper chamber and were incubated with 10 ng/mL IL-1 (R&D Systems Inc., Minneapolis, MN, USA). The lower chamber contained 20 ng/mL monocyte chemoattractant protein-1 (R&D Systems). Plates were incubated at 37 °C for 48 hours. The non-migrating cells on the upper surface were removed; the invading cells on the underside were fixed, stained with crystal violet, and observed under the microscope. The mean number of cells on the lower side of the surface was calculated from 5 randomly selected fields (magnification, 200X).

### TUNEL assay and immunofluorescent staining

To study apoptosis, we performed TUNEL assays using an *in-situ* cell death detection kit (Roche Applied Science, Indianapolis, IN, USA) according to the manufacturer’s instructions. For TUNEL assay and immunofluorescent co-staining, frozen sections of aorta were fixed with Cytofix (BD Biosciences), permeabilized with Perm/Wash (BD Biosciences), and blocked with 10% normal goat serum in PBS for 1 h. After TUNEL assay, sections were stained with anti-SM22-α antibody at 4 °C for overnight, followed by staining with an Alexa Fluor 488 goat anti-rabbit IgG antibody at room temperature for 1 h. Sections or cells were observed with a Leica SP5 confocal microscope (Leica Microsystems) or an Olympus immunofluorescence microscope. For each aorta or cell treatment condition, images from 5 randomly selected views were captured. For each image, the number of positive cells and total nuclei were quantified, and the percentage of positive cells was calculated.

### Cell culture and transfection

Human thoracic aortic SMCs (ATCC, Manassas, VA, USA) were cultured in smooth muscle media (Cell Applications, Inc. San Diego, CA, USA) with 10% fetal bovine serum (Thermo Fisher Scientific). SMCs were treated with tumour necrosis factor-α 100 ng/ml (Thermo Fisher Scientific), hydrogen peroxide (H_2_O_2_) 300 µM(Sigma-Aldrich), angiotensin II 100 nM(Sigma-Aldrich) or PA 0.3 mM (Sigma-Aldrich). SMCs were also treated with different dose of PA (0 mM, 0.1 mM, 0.2 mM, 0.3 mM, 0.4 mM, 0.5 mM). SMCs were transfected with 50 or 100 nM of ADAMTS-4 siRNA (Thermo Fisher Scientific) by using Lipofectamine (Thermo Fisher Scientific) according to the manufacturer’s instructions. Transfection efficiency was confirmed by means of western blot.

### Western blot analysis

Protein lysates from treated cells or aortic tissues were prepared as previously described^19^. Nuclear and cytoplasmic proteins were isolated using NE-PER Nuclear and Cytoplasmic Extraction Kit (Thermo Fisher Scientific), following the manufacturer’s instructions. Protein samples (15 µg per lane) were subjected to sodium dodecyl sulfate (SDS) polyacrylamide gel electrophoresis and were transferred to PVDF membranes. The membranes were blocked for 1 h in blocking solution comprising Tris-buffered saline containing 5% nonfat dried milk and 0.5% Tween 20 and then were incubated with a primary antibody against ADAMTS-4 PARP-1, cleaved PARP (Asp214) (9548, Cell Signaling), cleaved caspase-3 (9661, Cell Signaling), or versican degradation product. The blots were then washed with PBS with tween, incubated with horseradish peroxidase-conjugated anti-rabbit or anti-mouse secondary antibodies (Cell Signaling), and developed with Clarity Enhanced Chemiluminescence (ECL; Bio-Rad). The blots were exposed with HyBlot ES autoradiography film (Denville Scientific Inc., Holliston, MA) and quantified by using Image J Software (National Institutes of Health, Bethesda, MD). We confirmed equal protein loading by immunoblotting with HRP-conjugated β-actin antibody (Santa Cruz) or GAPDH antibody (Santa Cruz), β-tubulin, or lamin B1 (Cell Signaling).

### Co-immunoprecipitation analysis

Immunoprecipitation was performed as described previously^19^. Briefly, SMCs were lysed for 30 minutes in ice-cold extraction buffer containing 50 mM Tris-Cl (pH 7.5), 100 mM NaCl, 1% Triton X-100, 1 mM dithiothreitol, 1 mM EDTA, 1 mM EGTA, 2 mM Na3VO4, 50 mM β-glycerophosphate, and a protease inhibitor mixture (Amersham Biosciences, Little Chalfont, UK). The cell lysates were first precleared by incubating the lysate with IgG that was precoupled with protein A/G-agarose beads (Santa Cruz) at 4 °C for 90 minutes. The cleared lysate was then incubated with anti-ADAMTS-4 antibody conjugated with protein A/G-agarose beads at 4 °C overnight. The beads were washed twice with extraction buffer, twice with cell lysis buffer containing 0.5 M LiCl, and twice with cell lysis buffer. Proteins were eluted directly in SDS sample buffer for western blot analysis.

### Proteolysis of PARP-1 by ADAMTS-4

For *in vitro* proteolysis studies, 0.5 μg recombinant human PARP-1 (Life Technologies, Carlsbad, CA, USA) was incubated with 0.44 μg affinity-purified, recombinant human ADAMTS-4 (R&D Systems, Minneapolis, MN, USA) in reaction buffer (50 mM Tris, 10 mM CaCl_2_, pH 7.5) with a total volume of 25 μl at 37 °C for 1 to 3 hours. The broad-spectrum pan extracellular matrix protease inhibitor batimastat (Sigma-Aldrich; 0.125 mg/ml) was used to inhibit ADAMTS-4 activity. The products of PARP-1 digestion were analysed by western blot.

### Statistical analysis

All quantitative data are presented as the mean ± standard deviation. Data were analysed by using SPSS software, version 11.0 (SPSS Inc., Chicago, IL, USA). Normality of the data was examined by using the Kolmogorov-Smirnov test. Comparisons between two groups were performed by using independent t tests; multiple groups were compared by using one-way analysis of variance (ANOVA) or the Kruskal-Wallis test, as appropriate. P values were adjusted with Bonferroni method for pairwise comparisons when indicated. The incidence of aortic dissection or aneurysm was analysed by Fisher’s exact test. Kaplan-Meier survival curves were plotted to analyse the mouse survival rates, and the differences were analysed with the log-rank (Mantel–Cox) test. For all statistical analyses, 2-tailed probability values were used. A probability value of P < 0.05 was considered significant.

## Electronic supplementary material

Supplementary data
