# Supplementary material for: Critical Role of ADAMTS-4 in the Development of Sporadic Aortic Aneurysm and Dissection in Mice
Source: Sci Rep. 2017 Sep 27;7:12351. doi: 10.1038/s41598-017-12248-z (PMC5617887; doi:10.1038/s41598-017-12248-z)
Supplement: Supplementary file 1 — Supplementary data [file 41598_2017_12248_MOESM1_ESM.pdf]

Critical Role of ADAMTS-4 in the Development of Sporadic Aortic Aneurysm and Dissection in Mice

Pingping Ren[1†], Michael Hughes[1†], Swapna Krishnamoorthy[1], Sili Zou[1,3], Lin Zhang[1], Darrell Wu[1,7], Chen Zhang[1], John A. Curci[4], Joseph S. Coselli[1,2,6], Dianna M. Milewicz[5], Scott A. LeMaire[1,2,6,7*], & Ying H. Shen[1,2,6,*]

[1]Division of Cardiothoracic Surgery, Michael E. DeBakey Department of Surgery, Baylor College of Medicine, Houston, Texas, USA. [2]Department of Cardiovascular Surgery, Texas Heart Institute, Houston, Texas, USA. [3]Department of Vascular Surgery, Changzheng Hospital, Second Military Medical University, Shanghai, China. [4]Division of Vascular Surgery, Vanderbilt University School of Medicine, Nashville, Tennessee, USA. [5]Division of Medical Genetics, Department of Internal Medicine, The University of Texas Health Science Center at Houston, Houston, Texas, USA. [6]Cardiovascular Research Institute, Baylor College of Medicine, Houston, Texas, USA. [7]Department of Molecular Physiology and Biophysics, Baylor College of Medicine, Houston, Texas, USA.

[†]These authors contributed equally to the study.

[*]Correspondence and requests for materials should be addressed to Ying H. Shen, One Baylor Plaza, BCM 390, Houston, Texas 77030, USA. Phone 832-355-9952; fax 832-355-9951; e-mail hyshen@bcm.edu; and Scott LeMaire, MD, One Baylor Plaza, BCM 390, Houston, Texas 77030, USA. Phone 832-355-9942; fax 832-355-9948; e-mail slemaire@bcm.edu.

Figure S1

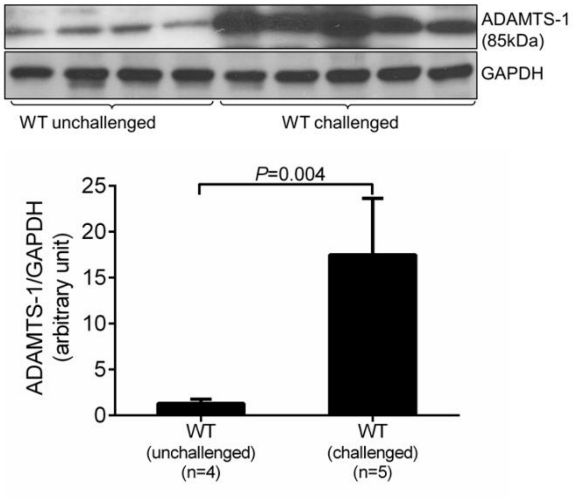

Figure S1. Representative western blot images of aortas from unchallenged WT mice (n=4) and challenged WT mice (n=5). Quantification studies showing the increased expression of ADAMTS-1 in the aortas of challenged WT mice versus unchallenged WT mice.

Figure S2

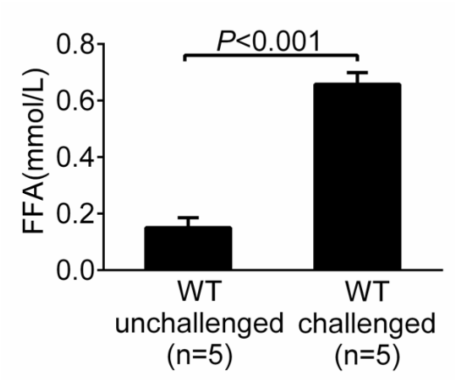

Figure S2. Free fatty acid levels in serum were significantly higher in challenged WT mice than in unchallenged WT mice.
